# Effect of rs4646994 polymorphism of angiotensin-converting enzyme on the risk of nonischemic cardiomyopathy

**DOI:** 10.1042/BSR20211617

**Published:** 2021-12-17

**Authors:** Jinsheng Shen, Xuesong Qian, Xiaofei Mei, Jialu Yao, Hezi Jiang, Kexin Li, Tan Chen, Yufeng Jiang, Yafeng Zhou

**Affiliations:** 1 Department of Cardiology, The First Affiliated Hospital of Soochow University, Suzhou City 215006, P.R. China; 2 Department of Cardiology, The First People's Hospital of Zhangjiagang city of Soochow University, Suzhou City, 215638, P.R. China; 3 Department of Cardiology, Dushu Lake Hospital Affiliated to Soochow University (Suzhou Dushu Lake Hospital), Suzhou City 215123, P.R. China

**Keywords:** ACE, DCM, HCM, meta-analysis, rs4646994

## Abstract

**Background:** Angiotensin-converting enzyme (ACE) gene polymorphisms have recently been shown to be associated with risk of developing left ventricular hypertrophy (LVH). However, the results were controversial. We aimed to conduct this meta-analysis to further confirm the association between ACE rs4646994 polymorphism and hypertrophic cardiomyopathy (HCM)/dilated cardiomyopathy (DCM).

**Methods:** PubMed, Embase, the Chinese National Knowledge Information, and Wanfang databases were searched for eligible studies. The Newcastle–Ottawa Scale (NOS) was used to evaluate the quality of included studies. Then we evaluated the association between ACE gene mutation and HCM/DCM by calculating odds ratios (ORs) and 95% confidence intervals (95% CIs). Subgroup analysis was further performed to explore situations in specialized subjects. Sensitivity analysis and publication bias was assessed to confirm the study reliability.

**Results:** There were 13 studies on DCM (2004 cases and 1376 controls) and 16 studies on HCM (2161 controls and 1192 patients). ACE rs4646994 polymorphism was significantly associated with DCM in all genetic models. However, in HCM, four genetic models (allele model, homozygous model, heterozygous model, and dominant model) showed significant association between ACE rs4646994 polymorphism and DCM. In subgroup analysis, we found that ACE rs4646994 polymorphism was significantly associated with DCM/HCM in Asian population. Finally, we also conducted a cumulative meta-analysis, which indicates that the results of our meta-analysis are highly reliable.

**Conclusion:** ACE rs4646994 polymorphism increases the risk of DCM/HCM in Asians, but not in Caucasians. More case–control studies are needed to strengthen our conclusions and to assess the gene–gene and gene–environment interactions between ACE rs4646994 polymorphism and DCM/HCM.

## Introduction

Cardiomyopathy is a kind of heterogeneous myocardial disease, resulting from pathological changes in the myocardium of different etiologies, manifesting as ventricular hypertrophy or dilation. Myocardial dysfunction due to other cardiovascular diseases is not part of the spectrum of the disease, such as valvular heart disease, hypertensive heart disease, congenital heart disease, coronary heart disease, or congenital heart disease [1,2]. It can eventually lead to progressive heart failure, arrhythmia, thromboembolism and sudden death, and has a poor prognosis. Cardiomyopathy can be generally classified into hypertrophic cardiomyopathy (HCM), dilated cardiomyopathy (DCM), restrictive cardiomyopathy (RCM), arrhythmogenic right ventricular cardiomyopathy (ARVC), and left ventricular noncompaction (LVNC). Among them, HCM and DCM are the main types of cardiomyopathy [3]. Many previous clinical studies recognized that cardiomyopathy has a familial origin, suggesting that genetic factors may play a crucial role in disease pathogenesis [4,5].

HCM and DCM are caused by mutant sarcomeric genes [6–8]. Mutations in sarcomeric protein genes can cause changes in myofilament tension that determine cardiac hypertrophy and dilation. Polymorphisms including genes encoding components of the renin–angiotensin system (RAS), such as angiotensin-converting enzyme (ACE), have recently been shown to be associated with the risk of developing left ventricular hypertrophy (LVH) [9,10] and thus may influence the clinical phenotype of HCM/DCM. The *ACE* gene is located on chromosome 17q23 and is characterized by a major insertion/deletion (rs4646994) polymorphism, consisting of a 289 base-pair Alu repeat sequence present or absent from intron 16 [11]. Past studies have shown that LVH is significantly increased in HCM and DCM patients with the ACE-D/D genotype and thus may be a genetic factor in the pathogenesis of HCM/DCM [12,13].

Over the past 20 years, numerous studies have reported the association of insertion/deletion polymorphisms of the angiotensin I-converting enzyme gene (ACE rs4646994) with HCM and DCM. But their results are inconsistent, especially the association with DCM, which is currently controversial [14]. Therefore, we conducted this meta-analysis to further confirm the association between ACE rs4646994 polymorphism and HCM/DCM.

## Methods

We followed Preferred Reporting Items for Systematic Reviews and Meta-Analyses (PRISMA) guidelines (http://prisma-statement.org/) in conducting the systematic review and meta-analysis.

### Search strategy

As of March 2021, we have used the terms ‘angiotensin converting enzyme’ or ‘ACE’, ‘polymorphism’ or ‘mutation’ and ‘hypertrophic cardiomyopathy’ and ‘dilated cardiomyopathy’ without language restrictions in PubMed, Embase, the Chinese National Knowledge Information, and Wanfang databases. Retrieved articles were reviewed to select related data of our interest. References included in the literature were also searched and reviewed to find other potentially eligible data.

### Inclusion criteria

The studies included in the meta-analysis must meet the following three criteria: (1) evaluating the association between ACE rs4646994 polymorphisms and HCM/DCM; (2) a case–control design was used, and (3) the data had to include the genotypes of II, ID, and DD, as well as comprehensive statistical indexes that were direct or indirect: odds ratio (OR) and 95% confidence interval (95% CI) and fulfilled the Hardy–Weinberg equilibrium (HWE) among the control groups.

### Exclusion criteria

All patients were excluded for potential influencing factors such as hypertension, hypertensive heart disease, coronary atherosclerotic heart disease, ischemic heart disease, ischemic cardiomyopathy, severe coronary obstruction for DCM valvular heart disease, valvular heart disease, congenital heart or vascular malformations, and inherent pulmonary disease.

### Data extraction

Two authors independently reviewed all the included studies and extracted vital data. Disagreements were resolved by a third researcher, and a common outcome was finally reached. We extracted the following information: first author, year of publication, country from which subjects came, ethnicity, number of cases and controls, allele and genotype frequencies, source of control group, diagnostic criteria, and HWE test. We attempted to contact the original authors if study data were incomplete. Study quality was assessed by the Newcastle–Ottawa Scale (NOS).

### Statistical methods

HWE was performed in the control group, and the significance level was set at *P*<0.05. The association between ACE rs4646994 polymorphisms and HCM/DCM was assessed by fixed- or random-effects models incorporating ORs and 95% CIs. We demonstrated the degree of heterogeneity between studies by using *I^2^* ranging from 0% (complete agreement) to 100% (complete inconsistency). We used a random-effects model (Der Simonian and Laird method) for the pooled analysis, and *I^2^* > 50% indicated heterogeneity among studies. Otherwise, a fixed-effects model (Mantel–Haenszel method) should be used. We also performed subgroup analysis to identify possible heterogeneity and cumulative meta-analysis to determine the reliability of the results. All analyses were performed in five genetic models: allelic model (d vs. I), homozygous model (DD vs. II), heterozygous model (ID vs. II), dominant model (ID + DD vs. II), and recessive model (DD vs. ID + II). Sensitivity analyses assessed the potential impact of individual study datasets on pooling or omitting studies. We also performed Egger’s test and plotted Begg’s funnel plot to determine publication bias, and concluded that there was no statistically significant publication bias when *P*>0.05. All statistical tests were performed using Stata version 15.0 (Stata Corp, University of Texas).

## Results

### Research characteristics

**Figure 1:**
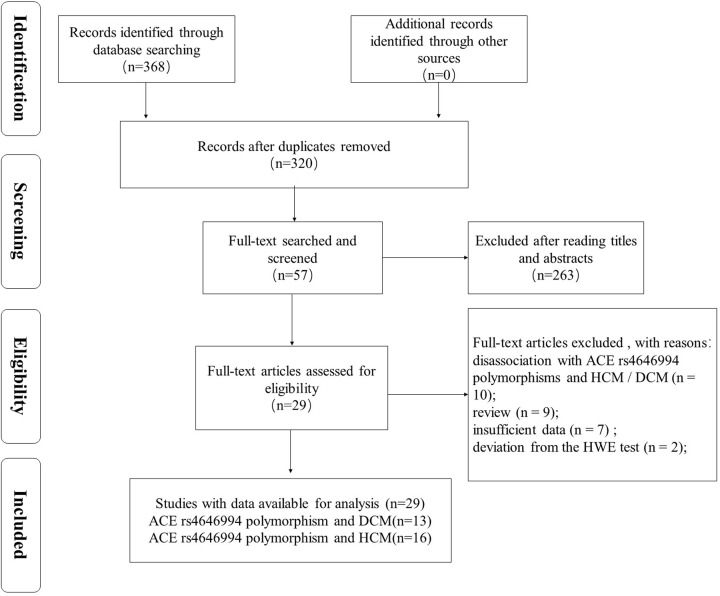
The PRISMA flow diagram of the study selection and exclusion

Finally we found a total of 368 potential articles related to keywords, of which 48 duplicate studies were excluded. We then initially screened the remaining 320 articles, 263 of which were excluded. From the full-text reading of 57 articles, 28 were excluded because of their disassociation with ACE rs4646994 polymorphisms and HCM/DCM (*n*=10), review (*n*=9), insufficient data (*n*=7), and deviation from the HWE test (*n*=2). The entire process of exclusion and enrollment is shown in Figure 1. Finally, this meta-analysis included 13 studies on DCM [13,15–26] (2004 controls and 1376 cases, Table 1) and 16 studies on HCM [13,17,20,24,27–38] (2161 controls and 1192 patients, Table 2). The NOS score of each study was more than 6, and therefore, the quality was good. The results are shown in Tables 1 and 2.

**Table 1:** The characteristics of included studies and ACE rs4646994 polymorphism genotype distribution and allele frequency of DCM in case and control groups

Author	Year	Country	Ethnicity	Sample size	Genotype (*n*)								Allele frequency (*n*, %)						NOS score	HWE test
					Cases				Controls				Cases			Controls				
					II	ID	DD	Total	II	ID	DD	Total	I	D	RAF	I	D	RAF		
Montgomery et al.	1995	U.K.	Caucasian	463	18	50	31	99	84	168	112	364	86	112	0.57	336	392	0.54	6	0.173
Sanderson et al.	1996	China	Asian	200	39	49	12	100	39	48	13	100	127	73	0.37	126	74	0.37	6	0.767
Yamada et al.	1997	Japan	Asian	210	36	35	17	88	50	55	17	122	107	69	0.39	155	89	0.36	6	0.764
Tiret et al.	2000	France	Caucasian	809	94	200	128	422	71	190	126	387	388	456	0.54	332	442	0.57	6	0.966
Shan et al.	2001	China	Asian	238	27	25	31	83	50	80	25	155	79	87	0.52	180	130	0.42	6	0.456
Wu et al.	2002	China	Asian	106	14	22	7	43	23	28	12	63	50	36	0.42	74	52	0.41	6	0.509
Zou et al.	2005	China	Asian	96	12	18	13	43	28	20	5	53	42	44	0.51	76	30	0.28	6	0.609
Rai et al.	2008	India	Asian	215	8	33	10	51	47	87	30	164	49	53	0.52	181	147	0.45	6	0.353
Kucukarabaci et al.	2008	Turkey	Caucasian	49	5	18	6	29	7	9	4	20	28	30	0.52	23	17	0.43	6	0.722
Mahjoub et al.	2010	Tunisia	Caucasian	227	12	38	26	76	46	83	22	151	62	90	0.59	175	127	0.42	6	0.116
Kong et al.	2012	China	Asian	206	20	49	32	101	30	53	22	105	89	113	0.56	113	97	0.46	6	0.874
Rani et al.	2017	India	Asian	377	15	120	42	177	72	86	42	200	150	204	0.58	230	170	0.43	6	0.089
Chen et al.	2017	China	Asian	184	17	29	18	64	51	57	12	120	63	65	0.51	159	81	0.34	6	0.496

Abbreviations: D, mutant type; I, wildtype; *n*, number; RAF, risk allele frequency; risk allele, D allele.

**Table 2:** The characteristics of included studies and ACE rs4646994 polymorphism genotype distribution and allele frequency of HCM in case and control groups

Author	Year	Country	Ethnicity	Sample size	Genotype (*n*)								Allele frequency (*n*, %)						NOS score	HWE test
					Cases				Controls				Cases			Controls				
					II	ID	DD	Total	II	ID	DD	Total	I	D	RAF	I	D	RAF		
Marian et al.	1993	U.S.A.	Caucasian	206	7	49	44	100	22	46	38	106	63	137	0.69	90	122	0.58	6	0.778
Yamada et al.	1997	Japan	Asian	193	31	32	8	71	50	55	17	122	94	48	0.34	155	89	0.36	6	0.667
Moiseev et al.	1997	Russia	Caucasian	181	2	5	6	13	33	55	80	168	9	17	0.65	121	215	0.64	6	0.315
Lopez-Haldon et al.	1999	Spain	Caucasian	309	2	13	25	40	33	125	111	269	17	63	0.79	191	347	0.64	6	0.952
Cai et al.	2000	China	Asian	101	16	16	13	45	26	23	7	56	48	42	0.47	75	37	0.33	6	0.528
Gao et al.	2000	China	Asian	101	12	15	13	40	31	18	12	61	39	41	0.51	80	42	0.34	6	0.185
Yang et al.	2000	China	Asian	149	13	35	15	63	37	36	13	86	61	65	0.52	110	62	0.36	6	0.86
Li et al.	2001	China	Asian	96	13	19	1	33	28	23	12	63	45	21	0.32	79	47	0.37	6	0.001
Ogimoto et al.	2002	Turkey	Caucasian	343	53	64	21	138	83	95	27	205	170	106	0.38	261	149	0.36	6	0.653
Zou et al.	2003	China	Asian	66	5	7	1	13	28	20	5	53	17	9	0.35	76	30	0.28	6	0.052
Kawaguchi et al.	2003	Japan	Asian	168	26	41	13	80	43	28	17	88	93	67	0.42	114	62	0.35	6	0.661
Doolan et al.	2004	Australia	Caucasian	236	10	14	12	36	48	94	58	200	34	38	0.53	190	210	0.52	6	0.147
Rai et al.	2008	India	Asian	282	11	63	44	118	47	87	30	164	85	151	0.64	181	147	0.45	6	0.048
Kaya et al.	2010	Turkey	Asian	83	8	34	21	63	5	9	6	20	50	76	0.6	19	21	0.52	6	0.661
Coto et al.	2010	Spain	Caucasian	507	35	100	72	207	46	135	119	300	170	244	0.59	227	373	0.62	6	0.147
Rani et al.	2017	India	Asian	332	16	89	27	132	72	86	42	200	121	143	0.54	230	170	0.43	6	0.048

Abbreviations: D, mutant type; I, wildtype; *n*, number; RAF, risk allele frequency; risk allele, D allele.

### Association between ACE rs4646994 polymorphism and susceptibility to DCM

**Figure 2:**
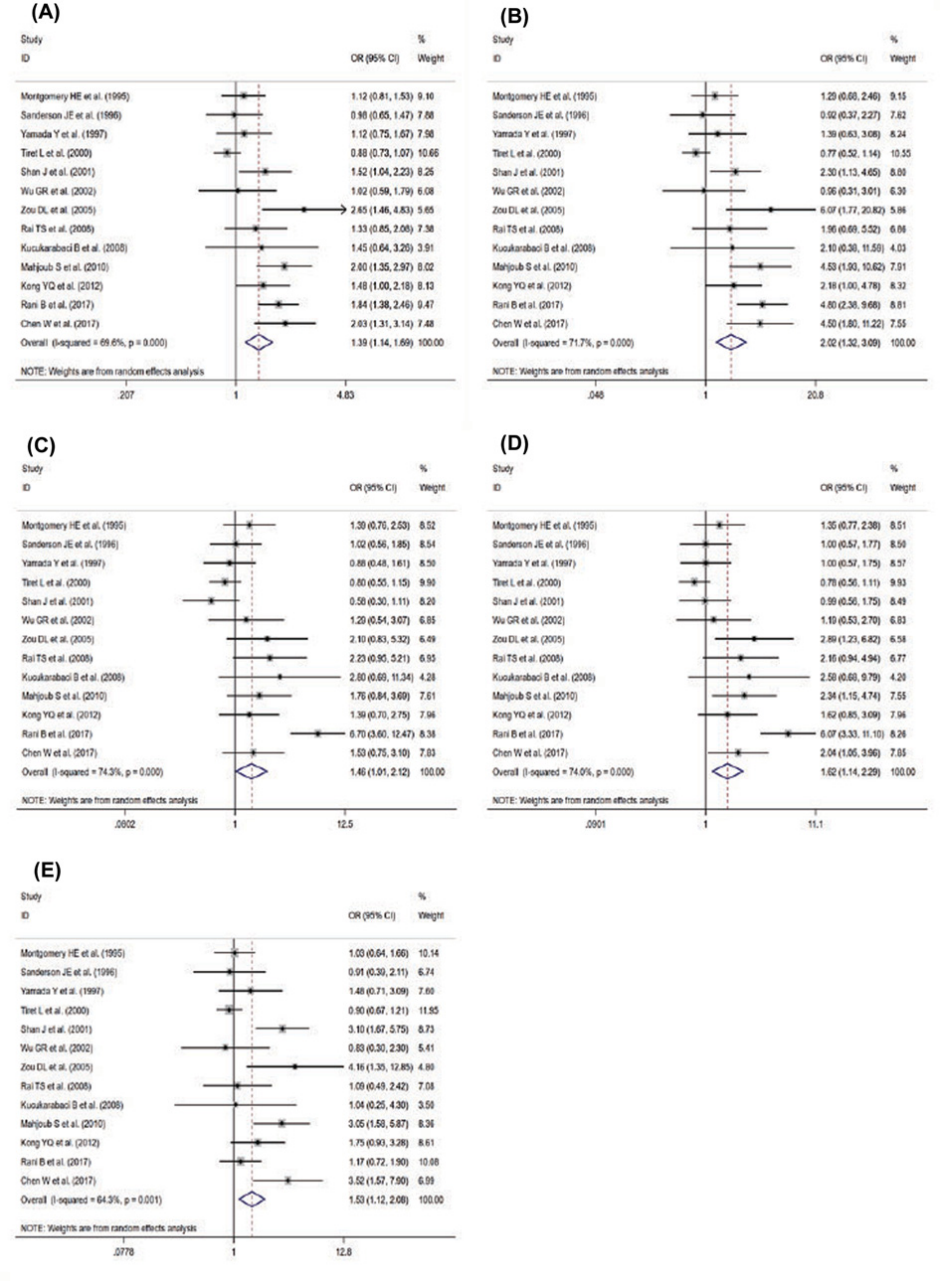
Forest plot from the meta-analysis on the association of ACE rs4646994 gene polymorphism and DCM risk (**A**) Allele; (**B**) homozygote; (**C**) heterozygote; (**D**) dominant; and (**E**) recessive.

Our meta-analysis showed that potential heterogeneity was found in all five genetic models (allele model: *I^2^*: 69.6%; homozygous gene model: *I^2^*: 71.7%; heterozygous gene model: *I^2^*: 74.3%; dominant gene model: *I^2^*: 74%; recessive gene model: *I^2^*: 64.3%). Therefore, a random-effects model was used in the meta-analysis (Figure 2). The results of the study on the association between ACE rs4646994 polymorphism and the pathogenesis of DCM showed that allele gene model (D vs. I): OR = 1.39, 95% CI = 1.14–1.69, *P*=0.001; homozygote gene model (DD vs. II): OR = 2.02, 95% CI = 1.32–3.09, *P*=0.001; heterozygote gene model (ID vs. II): OR = 1.46, 95% CI = 1.01–2.12, *P*=0.045; dominance gene model (ID+DD vs. II): OR = 1.62, 95% CI = 1.14–2.29, *P*=0.006; recessive gene model (DD vs. ID and II): OR = 1.53, 95% CI = 1.12–2.08, *P*=0.007. In summary, our meta-analysis showed that there was a significant association between ACE rs4646994 polymorphism and DCM in the five gene models. It can be concluded that the D allele and DD genotype of ACE rs4646994 polymorphism may be the genetic risk factors of DCM.

We try to determine more reliable results and explore the sources of heterogeneity by analyzing different subgroups. First of all, a subgroup analysis was carried out on the ethnicity (Asian race and White race). As shown in Table 3, the results show that four gene models of Asian race suggest that there is a significant association between ACE rs4646994 polymorphism and DCM (allele gene model: OR = 1.47, 95% CI = 1.21–1.78, *P*<0.001; homozygous gene model: OR = 2.28, 95% CI = 1.49–3.47, *P*<0.001; dominant gene model: OR = 1.72, 95% CI = 1.12–2.64, *P*=0.01; recessive gene model: OR = 1.67, 95% CI = 1.16–2.39, *P*=0.05). However, no association was shown between the Asian heterozygous gene model (heterozygous gene model: OR = 1.51, 95% CI = 0.91–2.50, *P*=0.11) and the white subgroup (allele gene model: OR = 1.25, 95% CI = 0.85–1.84, *P*=0.27; homozygous gene model: OR = 1.61, 95% CI = 0.70–3.67, *P*=0.26; Heterozygous gene model: OR = 1.27, 95% CI = 0.77–2.10, *P*=0.35; dominant gene model: OR = 1.40, 95% CI = 0.78–2.51, *P*=0.26; recessive gene model: OR = 1.29, 95% CI = 0.74–2.24, *P*=0.37). The results showed that the mutation of *ACE* gene significantly increased the risk of DCM in Asian population. Although there was no statistical significance between the mutation of ACE gene and the incidence of DCM in white population, it had a tendency to increase the risk of DCM. We conducted a subgroup analysis of the sample size, and the subgroup analysis of the sample size > 200 showed that there was an association between ACE rs4646994 polymorphism and the DCM risk of the three gene models (allele gene model: OR = 1.35, 95% CI = 1.07–1.70, *P*=0.01; homozygous gene model: OR = 1.97, 95% CI = 1.17–3.30, *P*=0.01; recessive gene model: OR = 1.47, 95% CI = 1.04–2.08, *P*=0.03). In the subgroup with sample size ≤ 200, this relationship disappeared (allele gene model: OR = 1.49, 95% CI = 1.00–2.23, *P*=0.05; homozygous gene model: OR = 2.16, 95% CI = 0.95–4.90, *P*=0.06; heterozygous gene model: OR = 1.40, 95% CI = 0.98–2.00, *P*=0.07; recessive gene model: OR = 1.66, 95% CI = 0.81–3.40, *P*=0.16).

**Table 3:** Subgroup analysis of association between ACE I/D gene polymorphism and DCM

	Number of studies	Allele comparison D vs. I					Homozygous DD vs. II					Heterozygous ID vs. II					Dominant ID + DD vs. II					Recessive DD vs. ID + II				
		OR	95% CI	*P*	*I^2^* (%)	*P* for *I^2^*	OR	95% CI	*P*	*I^2^* (%)	*P* for *I^2^*	OR	95% CI	*P*	*I^2^* (%)	*P* for *I*^2^	OR	95% CI	*P*	*I*^2^ (%)	*P* for *I*^2^	OR	95% CI	*P*	*I*^2^ (%)	*P* for *I*^2^
Total	13																									
Ethnicity																										
Asian	9	1.47	1.21–1.78	<0.001	48.5	0.05	2.28	1.49–3.47	<0.001	51.1	0.04	1.51	0.91–2.50	0.11	78.2	<0.001	1.72	1.12–2.64	0.01	73.7	<0.001	1.67	1.12–2.39	0.05	52.2	0.03
Caucasian	4	1.25	0.85–1.84	0.27	78.4	0.003	1.61	0.70–3.67	0.26	79.4	0.002	1.27	0.77–2.10	0.35	55.7	0.08	1.4	0.87–2.51	0.26	70.4	0.018	1.29	0.74–2.24	0.37	73	0.01
Sample size																										
>200	8	1.35	1.07–1.70	0.01	74	<0.001	1.97	1.17–3.30	0.01	77.2	<0.001	1.43	0.84–2.46	0.19	83.9	<0.001	1.6	0.99–2.59	0.06	82.4	<0.001	1.47	1.04–2.08	0.03	68.4	0.002
≤200	5	1.49	1.00–2.23	0.05	64.5	0.02	2.16	0.95–4.90	0.06	62	<0.001	1.4	0.98–2.00	0.07	0	0.584	1.62	1.06–2.49	0.03	32.8	<0.001	1.66	0.81–3.40	0.16	60	0.04

Abbreviation: I/D, insertion/deletion.

**Figure 3:**
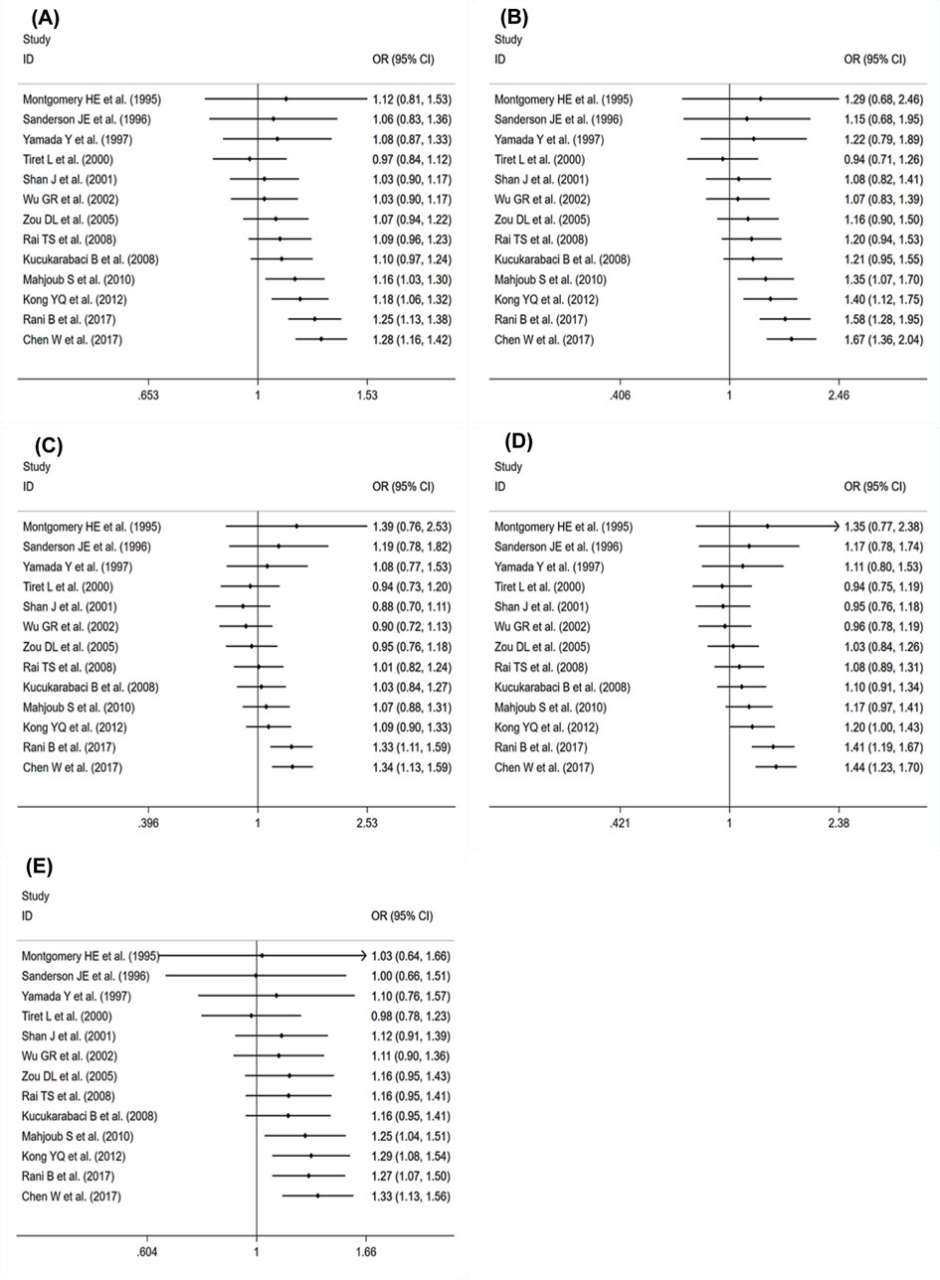
The cumulative meta-analysis of the association of ACE rs4646994 gene polymorphism and DCM risk (**A**) Allele; (**B**) homozygote; (**C**) heterozygote; (**D**) dominant; and (**E**) recessive.

In order to further determine the reliability of the results, through cumulative meta-analysis, we find that the more stable the association between ACE rs4646994 polymorphism and the incidence of DCM is as the year of publication approaches (Figure 3). It indicates that the results of this meta-analysis are very reliable.

### Association between ACE rs4646994 polymorphism and susceptibility to HCM

**Figure 4:**
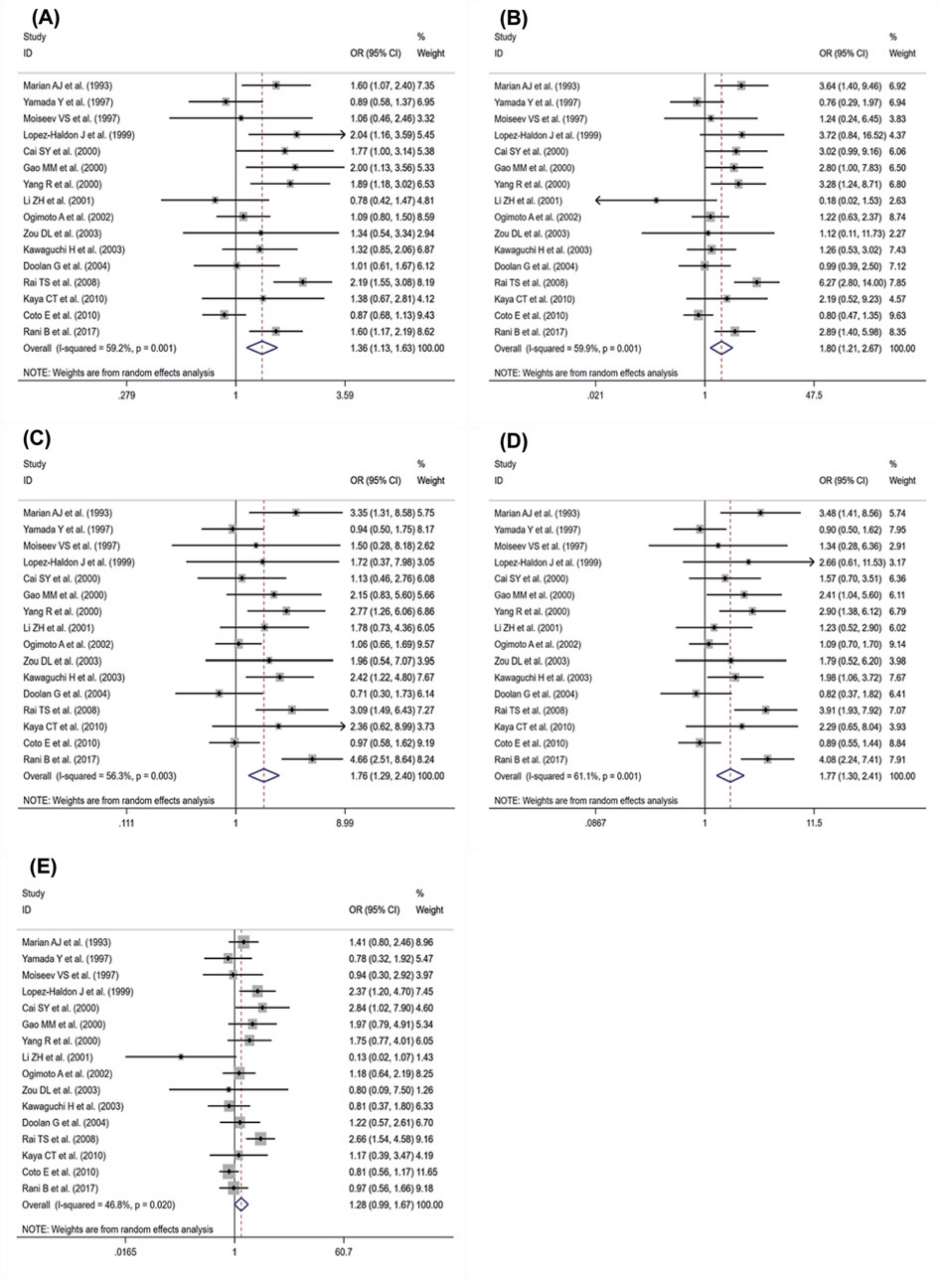
Forest plot from the meta-analysis on the association of ACE rs4646994 gene polymorphism and HCM risk (**A**) allele; (**B**) homozygote; (**C**) heterozygote; (**D**) dominant; and (**E**) recessive.

Our meta-analysis showed that there was a significant association between ACE rs4646994 polymorphism and HCM in four genetic models: allele gene model (D vs. I): OR = 1.36, 95% CI = 1.13–1.63, *P*=0.001; homozygous gene model (DD vs. II): OR = 1.80, 95% CI = 1.21–2.67, *P*=0.003; heterozygous gene model (ID vs. II): OR = 1.76, 95% CI = 1.29–2.40, *P*<0.001; dominant gene model (ID+DD vs. II): OR = 1.77, 95% CI = 1.30–2.41, *P*<0.001. The difference is that the recessive gene model (DD vs. ID and II: OR = 1.28, 95% CI = 0.99-1.67, *P*=0.064) shows that ACE gene mutation has nothing to do with HCM. However, the trend of increasing risk can still be seen. The results of the forest plot are shown in Figure 4.

In order to determine more reliable results and explore the source of heterogeneity, we conducted a subgroup analysis. First of all, we conducted a subgroup analysis of ethnicity, and Table 4 showed that the mutation of ACE gene was not associated with the incidence of HCM in White population (allele gene model: OR = 1.19, 95% CI = 0.91–1.54, *P*=0.02; homozygous gene model: OR = 1.40, 95% CI = 0.83–2.35, *P*=0.212; heterozygous gene model: OR = 1.18, 95% CI = 0.81–1.74, *P*=0.39; dominant gene model: OR = 1.25, 95% CI = 0.82–1.91, *P*=0.29; recessive gene model: OR = 1.21, 95% CI = 0.87–1.68, *P*=0.26). Although the recessive gene model (OR = 1.31, 95% CI = 0.87–1.97, *P*=0.20) analysis in Asian population showed that there was no association between ACE rs4646994 polymorphism and the incidence of HCM, there was a significant association among the other four models (allele gene model: OR = 1.49, 95% CI = 1.20–1.85, *P*<0.001; homozygous gene model: OR = 2.09, 95% CI = 1.25–3.50, *P*=0.005; heterozygous gene model: OR = 2.15, 95% CI = 1.51–3.06, *P*<0.001; dominant gene model: OR = 2.11, 95% CI = 1.48–3.00, *P*<0.001). Therefore, the mutation of ACE gene is associated with the increased incidence of HCM in Asian population. Then we made a subgroup analysis of the sample size. The results of subgroup analysis with sample size > 200 showed that there were three gene models suggesting that the mutation of ACE gene was associated with the pathogenesis of HCM (allele gene model: OR = 1.39, 95% CI = 1.05–1.84, *P*=0.02; homozygous gene model: OR = 2.06, 95% CI = 1.10–3.87, *P*=0.02; dominant gene model: OR = 1.89, 95% CI = 1.06–3.36, *P*=0.03), while the other two showed nothing to do with it (heterozygous gene model: OR = 1.78, 95% CI = 1.00–3.15, *P*=0.05; recessive gene model: OR = 1.36, 95% CI = 0.95–1.94, *P*=0.10). Similarly, the results in the subgroup with sample size ≤ 200 also showed that there was an association among the three gene models (allele gene model: OR = 1.33, 95% CI = 1.06–1.68, *P*=0.02; heterozygous gene model: OR = 1.71, 95% CI = 1.27–2.30, *P*<0.001; dominant gene model: OR = 1.71, 95% CI = 1.27–2.30, *P*<0.001), while there was no such phenomenon in the other two gene models (homozygous gene model: OR = 1.61, 95% CI = 0.99–2.64, *P*=0.06; recessive gene model: OR = 1.18, 95% CI = 0.78–1.79, *P*=0.44).

**Table 4:** Subgroup analysis of association between ACE I/D gene polymorphism and HCM

	Number of studies	Allele comparison D vs. I			I		Homozygous DD vs. I					Heterozygous ID vs. II					Dominant ID + DD vs. II					Recessive DD vs. ID + II				
		OR	95% CI	*P*	*I^2^* (%)	P for *I^2^*	OR	95% CI	*P*	*I^2^* (%)	*P* for *I^2^*	OR	95% CI	*P*	*I^2^* (%)	*P* for *I^2^*	OR	95% CI	*P*	*I^2^* (%)	*P* for *I^2^*	OR	95% CI	*P*	*I^2^* (%)	*P* for *I^2^*
Total	16																									
Ethnicity																										
Asian	10	1.49	1.20–1.85	<0.001	47	0.05	2.09	1.25–3.50	0.005	55.3	0.02	2.15	1.51–3.06	<0.001	45.3	0.06	2.11	1.48–3.00	<0.001	51.7	0.03	1.31	0.87–1.97	0.20	51.4	0.03
Caucasian	6	1.19	0.91–1.54	0.20	55.3	0.05	1.4	0.83–2.35	0.212	49.8	0.08	1.18	0.81–1.74	0.39	28.3	0.22	1.25	0.82–1.91	0.29	44.3	0.11	1.21	0.87–1.68	0.26	40.3	0.14
Sample size																										
>200	7	1.39	1.05–1.84	0.02	76.4	<0.001	2.06	1.10–3.87	0.02	76.9	<0.001	1.78	1.00–3.15	0.05	77.8	<0.001	1.89	1.06–3.36	0.03	80.3	<0.001	1.36	0.95-1.94	0.10	64.9	0.01
≤200	9	1.33	1.06–1.68	0.02	29.5	0.18	1.61	0.99–2.64	0.06	30.7	0.17	1.71	1.27–2.30	<0.001	0	0.50	1.65	1.25–2.19	<0.001	2.8	0.41	1.18	0.78-1.79	0.44	28.3	0.19

Abbreviation: I/D, insertion/deletion.

**Figure 5:**
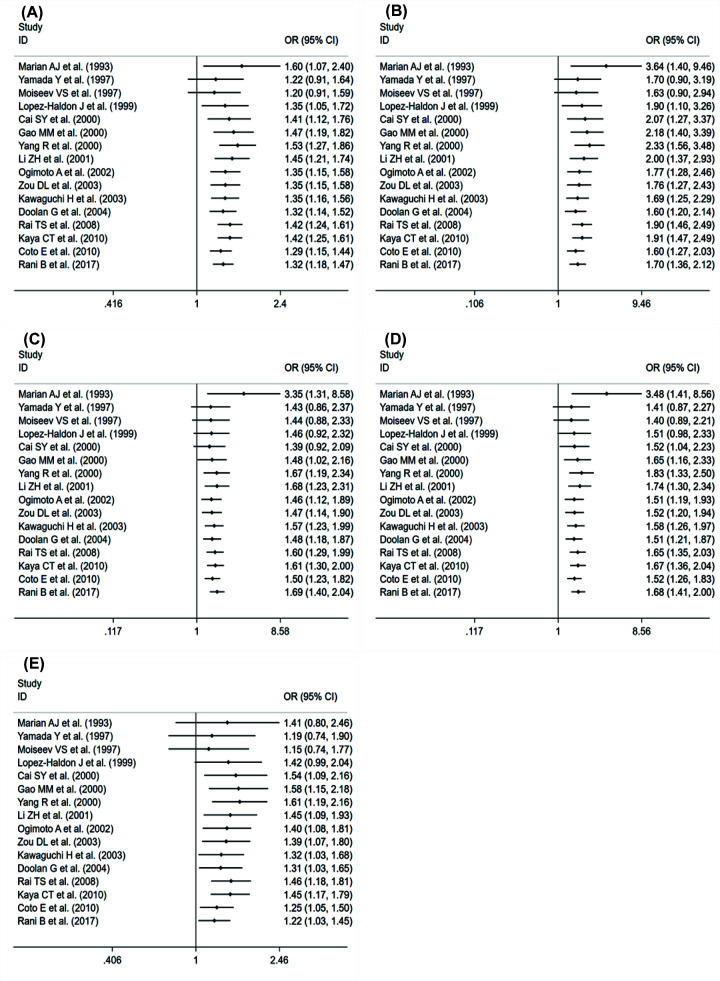
The cumulative meta-analysis on the association of ACE rs4646994 gene polymorphism and HCM risk (**A**) Allele; (**B**) homozygote; (**C**) heterozygote; (**D**) dominant; and (**E**) recessive.

As shown in Figure 5, through cumulative meta-analysis, we found that with the passage of time of the five gene models, the more stable the association between ACE rs4646994 polymorphism and the risk factors of HCM.

### Sensitivity analysis

We conducted the sensitivity analysis to assess whether omitting each study would change the overall ORs. As shown in Supplementary Figures S1 and S2, none of the studies would change the results of our meta-analysis, which showed that our results were reliable.

### Publication bias

The Begg’s funnel plots associated with the above analyses are presented in Supplementary Figure S3 and S4. From the Begg’s funnel plot, it can be seen that there was no obvious asymmetry in each meta-analysis, thus indicating that there was no publication bias in our study. We performed Egger’s test to further validate the above conclusion (DCM: allele model: *P*=0.068; homozygote model: *P*=0.051; heterozygote model: *P*=0.121; dominant model: *P*=0.040; and recessive model: *P*=0.079. HCM: allele model: *P*=0.472; homozygote model: *P*= 0.678; heterozygote model: *P*=0.343; dominant model: *P*=0.087; and recessive model: *P*=0.897).

## Discussion

In this meta-analysis, we critically reviewed all eligible published studies that met the inclusion and exclusion criteria to evaluate the association between ACE rs4646994 polymorphisms and the risk of DCM/HCM. There were 13 studies regarding DCM and 16 on HCM. Our findings suggest that ACE rs4646994 polymorphisms may be associated with both HCM and DCM.

Polymorphisms in the ACE gene, encoding one of the components of the renin–angiotensin–aldosterone system (RAAS), have been found to be associated with a variety of cardiovascular diseases, such as hypertension, myocardial infarction, and cardiomyopathy [39,40]. Increased synthesis of angiotensin II induces cell proliferation, migration, and hypertrophy and can enhance proinflammatory cytokine and matrix metalloproteinase production. Studies have shown that the anatomical features of HCM are characterized by asymmetric hypertrophy of the ventricles, whereas DCM is a class of cardiomyopathies characterized by systolic dysfunction of the left ventricle or biventricular enlargement class. Meanwhile, aldosterone production is regulated by the renin–angiotensin system, and studies have shown that it has a direct effect on the heart, including recurrent cardiac hypertrophy and fibrosis, ultimately leading to cardiac remodeling [41,42]. Therefore, ACE rs4646994 polymorphisms provisionally play an important role in the pathogenesis of HCM/DCM cardiomyopathy. While the results of our meta-analysis revealed that ACE rs4646994 polymorphism was associated with the risk of DCM/HCM incidence, providing a rationale of genetic aspects for the treatment of DCM/HCM.

Our subgroup analysis showed that ACE gene mutations can increase the risk of DCM and HCM in Asian population, while no such results were obtained for Caucasian population. The above results suggest an association between the risk of incident DCM and HCM and the race. In addition, the population may be the source of heterogeneity because the heterogeneity was reduced in the subgroup analysis of the population. In the subgroup analysis with a dividing line of sample size 200, we found that the association of ACE gene mutations with the risk of DCM incidence was shown in the subgroup analysis with a sample size greater than 200 in DCM but not in the subgroup analysis with less than 200. Therefore, we believe that a larger sample size is needed for the association of DCM with ACE gene mutations to confirm the reliability of the results. Whereas in HCM the analysis of a subsample size, both showed an association of ACE gene mutations with the onset of HCM. The results of our time-series analyses all showed a stable relationship between ACE gene mutations and the risk of incident DCM/HCM. None of the studies could change the meta-analysis results in the sensitivity analysis (Supplementary Figures S1 and S2). No publication bias was found in our study (Supplementary Figures S3 and S4).

There are certain limitations to our study. First, we failed to group familial DCM/HCM and sporadic DCM/HCM due to limited data. Second, most of our reference studies had small sample sizes, which may affect the results of the meta-analysis. Third, the ethnic distribution of our included studies was relatively single, only Caucasian and Asian ethnicities were included, and subgroup analysis could not be performed for all ethnic populations. Besides, heterogeneity due to differences in the regression models of the included studies could not be avoided due to unavailability of specific information. The review protocol of the present study was not pre-registered with PROSPERO.

## Conclusion

This meta-analysis showed an association between the onset of HCM/DCM and ACE rs4646994 polymorphism. The findings of the current study may contribute to stratification strategies for patients with HCM/DCM. In addition, these results also show the potential possibility to treat HCM/DCM by modulating the RAAS system function in patients.

## Supplementary Material

Supplementary Figures S1-S4

## Data Availability

The datasets used and/or analyzed during the current study are available from the corresponding authors on reasonable request.

## Abbreviations

ACE: angiotensin-converting enzyme
DCM: dilated cardiomyopathy
HCM: hypertrophic cardiomyopathy
HWE: Hardy–Weinberg equilibrium
LVH: left ventricular hypertrophy
NOS: Newcastle–Ottawa Scale
OR: odds ratio
RAAS: renin–angiotensin–aldosterone system
95% CI: 95% confidence interval
